# A lipidomics study reveals hepatic lipid signatures associating with deficiency of the LDL receptor in a rat model

**DOI:** 10.1242/bio.019802

**Published:** 2016-07-04

**Authors:** Hong Yu Wang, Chao Quan, Chunxiu Hu, Bingxian Xie, Yinan Du, Liang Chen, Wei Yang, Liu Yang, Qiaoli Chen, Bin Shen, Bian Hu, Zhihong Zheng, Haibo Zhu, Xingxu Huang, Guowang Xu, Shuai Chen

**Affiliations:** 1 State Key Laboratory of Pharmaceutical Biotechnology and MOE Key Laboratory of Model Animal for Disease Study, Model Animal Research Center, Nanjing University, Pukou District, Nanjing 210061, China; 2 Collaborative Innovation Center of Genetics and Development, Shanghai 200438, China; 3 CAS Key Laboratory of Separation Science for Analytical Chemistry, Dalian Institute of Chemical Physics, Chinese Academy of Sciences, 457 Zhongshan Road, Dalian 116023, China; 4 Laboratory Animal Center, China Medical University, Shenyang 110001, China; 5 State Key Laboratory of Bioactive Substances and Functions of Natural Medicines, Institute of Materia Medica, Chinese Academy of Medical Sciences and Peking Union Medical College, Beijing 100050, People's Republic of China

**Keywords:** Low-density lipoprotein receptor (LDLR), Cholesterol, Lipid metabolism, Rat, Knockout

## Abstract

The low-density lipoprotein receptor (LDLR) plays a critical role in the liver for the clearance of plasma low-density lipoprotein (LDL). Its deficiency causes hypercholesterolemia in many models. To facilitate the usage of rats as animal models for the discovery of cholesterol-lowering drugs, we took a genetic approach to delete the LDLR in rats aiming to increase plasma LDL cholesterol (LDL-C). An LDLR knockout rat was generated via zinc-finger nuclease technology, which harbors a 19-basepair deletion in the seventh exon of the *ldlr* gene. As expected, deletion of the LDLR elevated total cholesterol and total triglyceride in the plasma, and caused a tenfold increase of plasma LDL-C and a fourfold increase of plasma very low-density lipoprotein (VLDL-C). A lipidomics analysis revealed that deletion of the LDLR affected hepatic lipid metabolism, particularly lysophosphatidylcholines, free fatty acids and sphingolipids in the liver. Cholesterol ester (CE) 20:4 also displayed a significant increase in the LDLR knockout rats. Taken together, the LDLR knockout rat offers a new model of hypercholesterolemia, and the lipidomics analysis reveals hepatic lipid signatures associating with deficiency of the LDL receptor.

## Abstract (precis)

**Summary:** An LDL receptor knockout rat model was generated which offers a new hypercholesterolemia model. A lipidomics analysis reveals hepatic lipid signatures associating with LDLR deficiency in rats.

## INTRODUCTION

As a vital lipid in the body, cholesterol not only maintains the structural integrity and fluidity of cellular membranes but also functions as an important precursor for the biosynthesis of bile acids and steroid hormones. However, cholesterol levels have to be maintained tightly, and too much cholesterol (hypercholesterolemia) increases the risks of atherosclerosis and coronary heart disease (Narverud et al., 2014). Cholesterol, together with triacylglycerides (TAG) and other fats, is packed with apolipoprotein B and additional ancillary proteins to form large lipoprotein particles that transport lipids through the circulation. There are five types of lipoproteins according to their sizes, i.e. chylomicrons, very low-density lipoprotein (VLDL), intermediate-density lipoprotein (IDL), low-density lipoprotein (LDL) and high-density lipoprotein (HDL), among which LDL increases while HDL decreases the risks of atherosclerosis and coronary heart disease (Brunham and Hayden, 2015; Ishibashi et al., 1994a). The clearance of plasma LDL is mediated by the LDL receptor (LDLR) (Brown and Goldstein, 1986; Ishibashi et al., 1993), whose deficiency in human causes a massive elevation of plasma TC (600-1000 mg/dl) and consequently leads to fulminant atherosclerosis (Goldstein and Brown, 1989).

Cholesteryl ester transfer protein (CETP) is an important factor in regulating lipoprotein metabolism, which transfers cholesterol ester (CE) from HDL to lipoproteins of lower density and is closely linked to atherosclerosis (Masson et al., 2009; Quintao and Cazita, 2010). Some vertebrate species can be defined as two groups that have a distinct plasma lipoprotein profile and CETP activity (Liu et al., 2010, 1995; Tsutsumi et al., 2001). One group, including human, monkey, rabbit and hamster, has a low ratio of plasma HDL cholesterol (HDL-C) to total cholesterol (TC) and concomitant high CETP activity. In contrast the other group, including mouse, rat, dog and tree shrew, displays a high HDL-C/TC ratio and concomitant low CETP activity. As a consequence, the first group is susceptible to atherosclerosis while the second group exhibits resistance to atherosclerosis (Liu et al., 2010, 1995; Tsutsumi et al., 2001). The plasma lipoprotein profile in mice and rats hampers their usage as animal models for the discovery of cholesterol-lowering drugs. This plasma lipoprotein profile can be altered in mice through cholesterol-rich diet feeding or manipulation of the LDLR. For example, knockout of the LDLR in mice increases plasma LDL cholesterol (LDL-C) and causes hypercholesterolemia (Brown and Goldstein, 1986; Ishibashi et al., 1993). A cholesterol-rich diet can further increase plasma TC levels and shifts the cholesterol distribution further towards lipoproteins of lower density in the LDLR knockout mice (Ishibashi et al., 1994a).

Rats have been widely used as a model organism over the last 100 years with accumulation of a large volume of physiology data. The size of rats renders surgical procedures easier in this animal model than in mice, and also enables serial blood draws which make rats a useful model particularly for cardiovascular and metabolic diseases (Iannaccone and Jacob, 2009). However, the current way to elevate plasma LDL-C in rats with cholesterol-rich diet feeding, is not very effective (Vasant et al., 2014), which limits their usage for drug discovery to combat hypercholesterolemia.

It has been repeatedly demonstrated in various models that LDLR plays an important role in the liver in mediating the clearance of plasma LDL (Goldstein and Brown, 1989; Ishibashi et al., 1993; Watanabe, 1980). However, impacts of its deficiency on hepatic lipid metabolism have not been fully understood.

In this study, we aimed to take a genetic approach through deleting the LDLR to induce hypercholesterolemia in rats and to examine impacts of its deficiency on hepatic lipid metabolism. To this end, we generated a knockout rat that has a 19-basepair deletion in the seventh exon of the *ldlr* gene, and carried out a lipodomics analysis of the liver of this LDLR knockout rat.

## RESULTS

### Generation and molecular characterization of the LDLR knockout rat

**Fig. 1.**
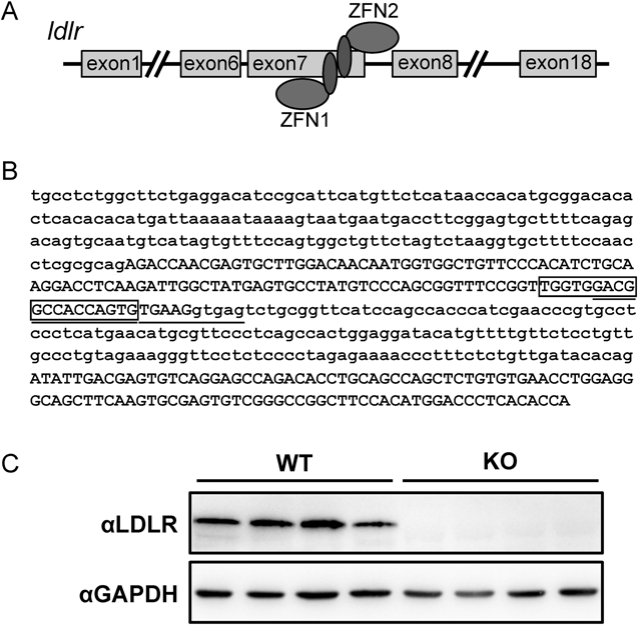
**Generation of the LDLR knockout rats.** (A) Illustrative diagram of the strategy for generation of the LDLR knockout rats. Sequence information of ZFN1 and ZFN2 targeting the *ldlr* gene is in Fig. S1. (B) The sequence information of the *ldlr* knockout allele. The 24-nucleotide underlined is the target sequence of the *ldlr*-ZFNs. The 19-nucleotide in the box was deleted in the LDLR knockout rats. (C) The LDLR protein expression in the LDLR knockout rats.

We designed a pair of zinc-finger nucleases (ZFNs) (Fig. S1) targeting a sequence (GACGGCCACCAGTGTGAAGGTGA) spanning the seventh exon and intron of the *ldlr* gene to generate an LDLR knockout rat as illustrated in Fig. 1A. The pair of ZFNs targeting the *ldlr* gene were *in vitro* transcribed into mRNAs that were subsequently injected into rat zygotes. Five hundred rat zygotes were injected and subsequently implanted into uterus of pseudo-pregnant rats. Twenty-two pups were born, among which six animals harbored mutations in the *ldlr* gene. Three of the mutant lines had a 2-bp deletion mutation in the sixth intron of the *ldlr* gene while the other three mutant lines contained deletion/insertion mutations in the seventh exon of the *ldlr* gene. The line No. 1 harbored a 19-bp deletion in the seventh exon of the *ldlr* gene (Fig. 1B), which could cause a shift in the reading frame. We further confirmed that the LDLR proteins were not detectable in liver lysates from the homozygous offspring of this line No. 1 (Fig. 1C), showing that this line is a LDLR knockout line. Therefore, this line No. 1 was renamed as the LDLR knockout rat and used in the following studies. This LDLR knockout rat was viable and displayed no overt phenotype within the experimental duration (20 weeks).

### The LDLR knockout rat displays hypercholesterolemia and hypertriglyceridemia

**Fig. 2.**
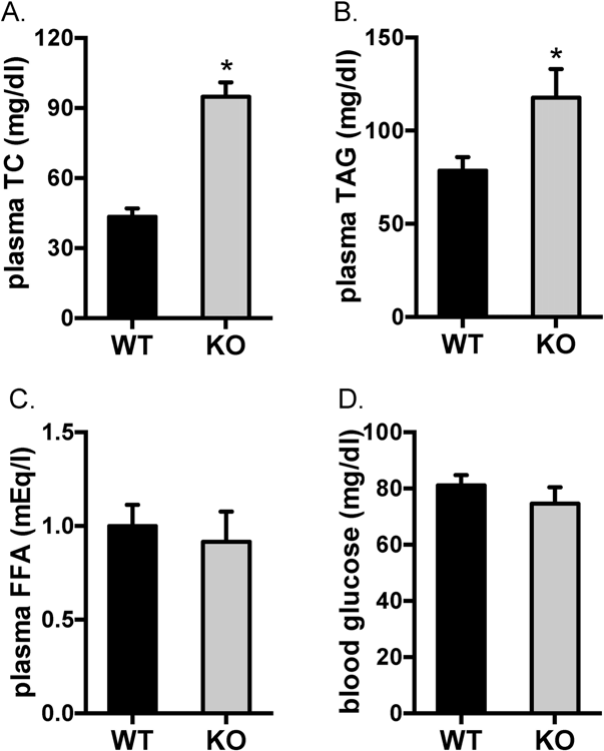
**Levels of plasma TC, TAG, FFA and blood glucose in the LDLR knockout rats.** Blood glucose, total cholesterol (TC), triacylglycerides (TAG) and free fatty acids (FFA) in the plasma were determined in 20-week-old rats. The data are given as the mean±s.e.m.; *P<0.05. WT: *n*=7; KO: *n*=5.

The LDLR is responsible for removal of LDL-C from the circulation. We therefore measured parameters of blood chemistry in the LDLR knockout rats. As expected, TC levels in the blood were significantly elevated by more than twofold in the LDLR knockout rats as compared to those of the wild-type controls (Fig. 2A). Total TAG levels were also significantly increased by ∼50% in the blood from the LDLR knockout rats as compared to those of the wild-type controls (Fig. 2B). In spite of hypercholesterolemia and hypertriglyceridemia in the LDLR knockout rats, the levels of blood glucose and plasma free fatty acids (FFA) were unaltered in these animals (Fig. 2C-D).

**Fig. 3.**
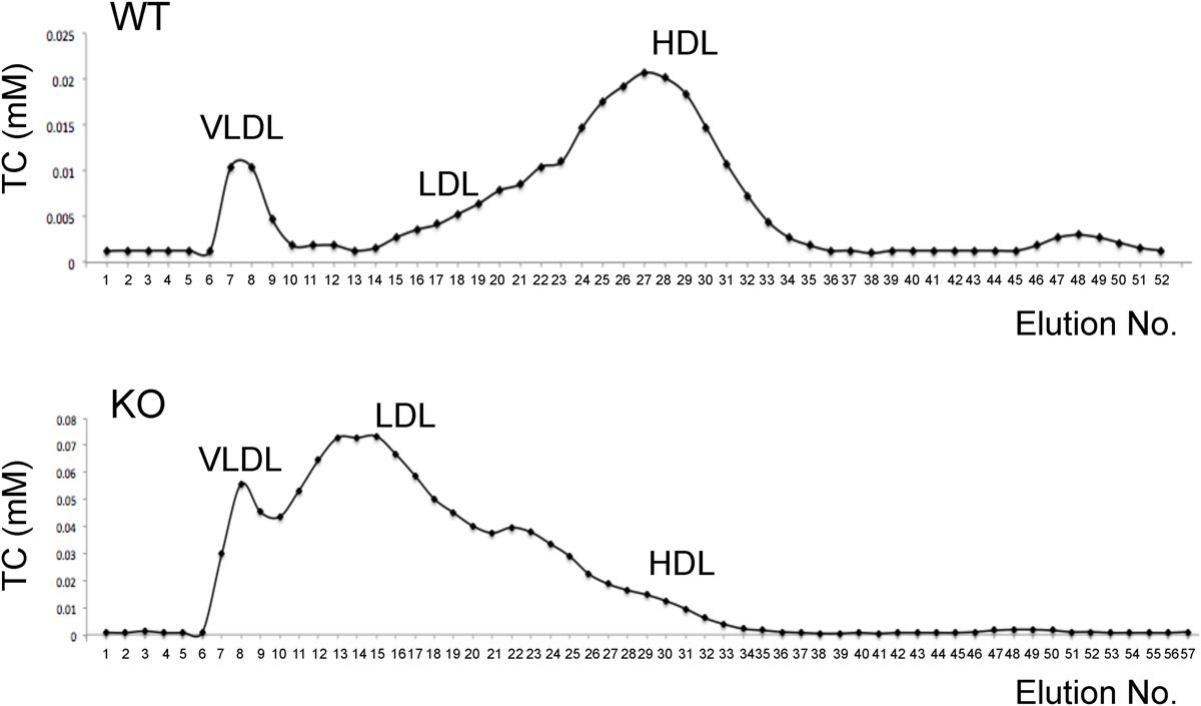
**Plasma lipoprotein profile in the LDLR knockout rats.** Lipoproteins in rat plasma were separated as described in the Materials and methods, and cholesterol content in each fraction was subsequently determined. Representative cholesterol distributions in lipoproteins for the wild-type and LDLR knockout rats were shown, and the quantitative data is shown in Table 1.

We next determined the cholesterol levels in different lipoprotein particles to get more insight into how deletion of the LDLR impacts on cholesterol metabolism in rat. As previously reported (Tsutsumi et al., 2001), HDL-C predominates in the plasma of wild-type rats whereas LDL-C and VLDL-cholesterol (VLDL-C) are much less abundant than HDL-C (Fig. 3 and Table 1). Deletion of the LDLR caused a drastic shift in cholesterol distribution among different lipoprotein particles in the plasma of rats. The LDL-C levels in the plasma had a more than tenfold increase, and the levels of plasma VLDL-C were elevated by more than fourfold in the LDLR knockout rats (Fig. 3 and Table 1). There was also a small but significant increase in the levels of plasma HDL-C in the LDLR knockout rats (Fig. 3 and Table 1).

**Table 1.**
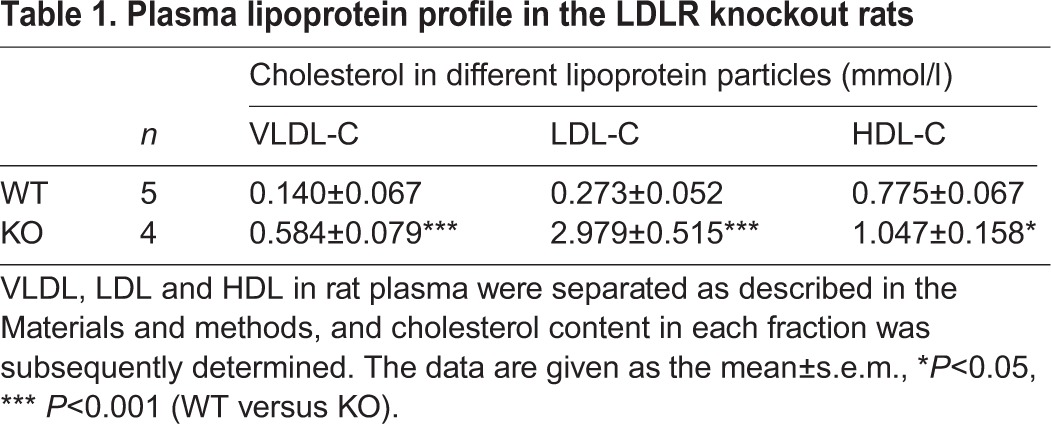
**Plasma lipoprotein profile in the LDLR knockout rats**

### Lipid profile is altered in the liver of the LDLR knockout rat

**Fig. 4.**
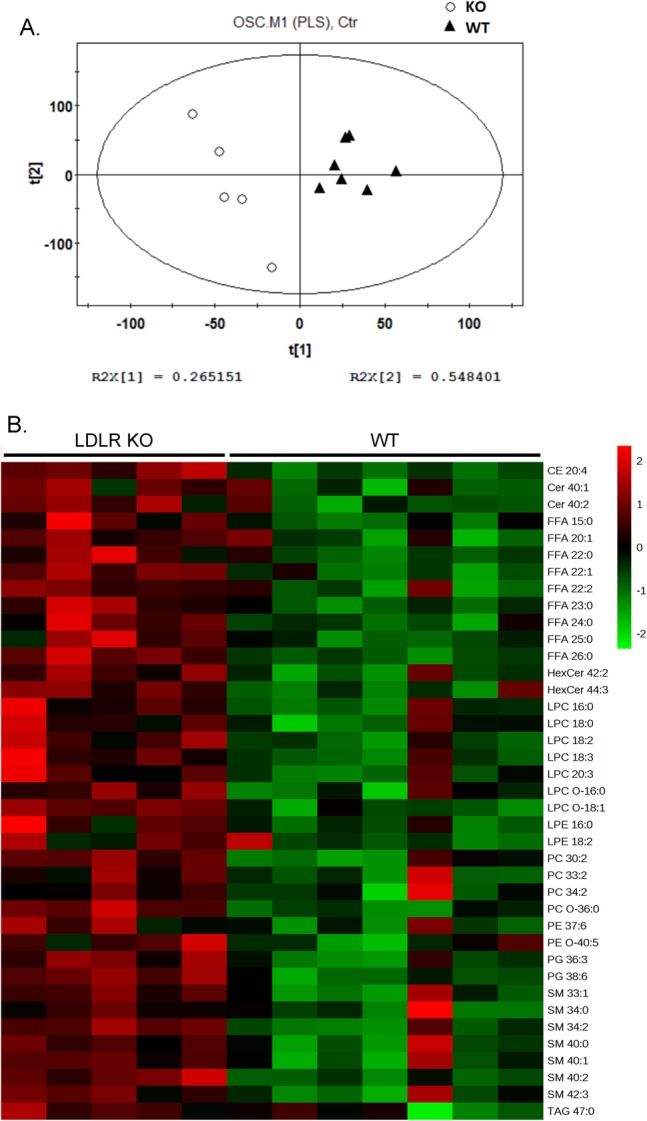
**Hepatic lipid profile in the LDLR knockout rats.** Lipids were extracted from the liver and determined via LC-MS. The levels of each lipid species in the wild-type rats were set to one, and their levels in the LDLR knockout rats were normalized against the respective wild-type values. (A) A partial least squares discriminant analysis after orthogonal signal correction (OSC-PLS-DA) of the data. (B) Heat map of the 39 significantly changed lipid species between the wild-type and LDLR knockout rats. The colors from green to red indicate the relative contents of lipid species in the LDLR knockout rats compared with those in the wild-type rats. WT: *n*=7; KO: *n*=5.

To investigate how deletion of the LDLR affects hepatic lipid metabolism, we next carried out a lipidomics study via liquid chromatography coupled to mass-spectrometry (LC-MS) in the liver of the LDLR knockout rats. We included eight lipid molecular species (Cer 17:0, TG 45:0, PC 38:0, PE 34:0, LPC 19:0, SM 12:0, FFA 16-d3, FFA 18-d3) from seven lipid classes as internal standards to evaluate the reproducibility and robustness of our methodology. The relative standard deviation ranged from 5.5 to 12.3% for the eight internal standards that were regularly inserted in the analytical sequence in the twelve LC-MS runs (Table S1), suggesting good repeatability for our method. In total, 295 lipid species were detected and identified in liver extracts through this method. A partial least squares discriminant analysis after orthogonal signal correction (OSC-PLS-DA) showed good separation of the two genotypes at the principal component 1 direction (Fig. 4A). We then carried out a univariate analysis on the lipidomics data. Among 295 lipid species analysed, 39 lipid species were significantly increased while none were decreased in the liver of the LDLR knockout rats (Fig. 4B), and details of the lipidomics data are described in the following sections.

### CE 20:4 among neutral lipids is specifically increased in the liver of the LDLR knockout rat

**Fig. 5.**
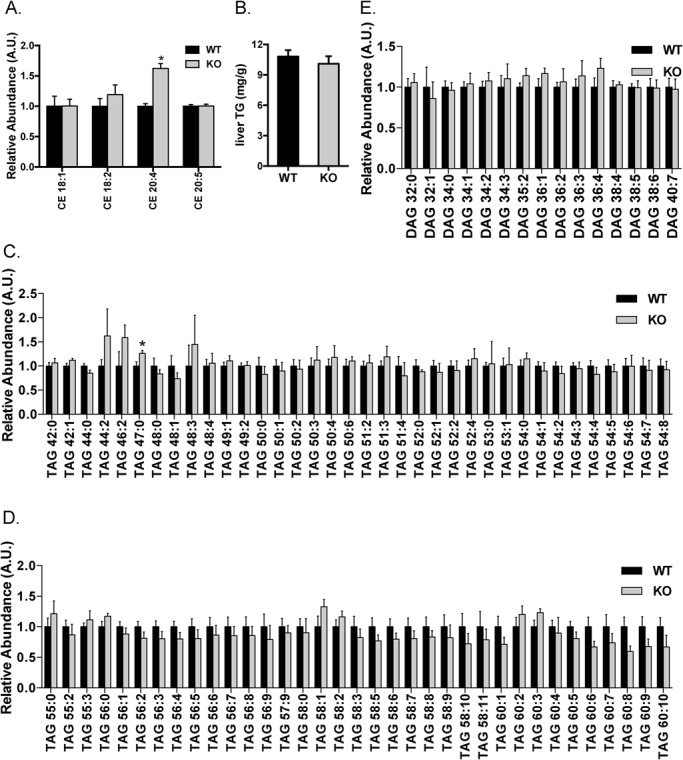
**Hepatic profiles of neutral lipids in the LDLR knockout rats.** Lipids were extracted from the liver, and CE (A), TAG (C,D) and DAG (E) species were determined via LC-MS. The levels of each lipid species in the wild-type rats were set to one, and their levels in the LDLR knockout rats were normalized against the respective wild-type values. Total TG levels in the liver were also determined using an enzymatic method (B). The data are given as the mean±s.e.m., *P<0.05. WT: *n*=7; KO: *n*=5.

Four CE species were detected via lipidomics in the liver, among which only CE 20:4 displayed a significant increase (>60%) in the liver of the LDLR knockout rats (Fig. 5A). LDL particles also carry substantial amount of TAG into the liver via LDLR. Despite the elevated TAG levels in the blood and deficiency of LDLR, the total TAG levels in the liver were normal in the LDLR knockout rats as compared to those in the wild-type controls when an enzymatic method was employed (Fig. 5B). Furthermore, the lipidomics analysis showed that all TAG species except one (TAG 47:0) detected in this study were comparable in the livers of the LDLR knockout and wild-type rats (Fig. 5C-D). Similarly, all digacylglyceride (DG) species detected in the lipidomics assay were normal in the liver of the LDLR knockout rats (Fig. 5E).

### Differential changes in phospholipids and free fatty acids (FFA) in the liver of the LDLR knockout rat

**Fig. 6.**
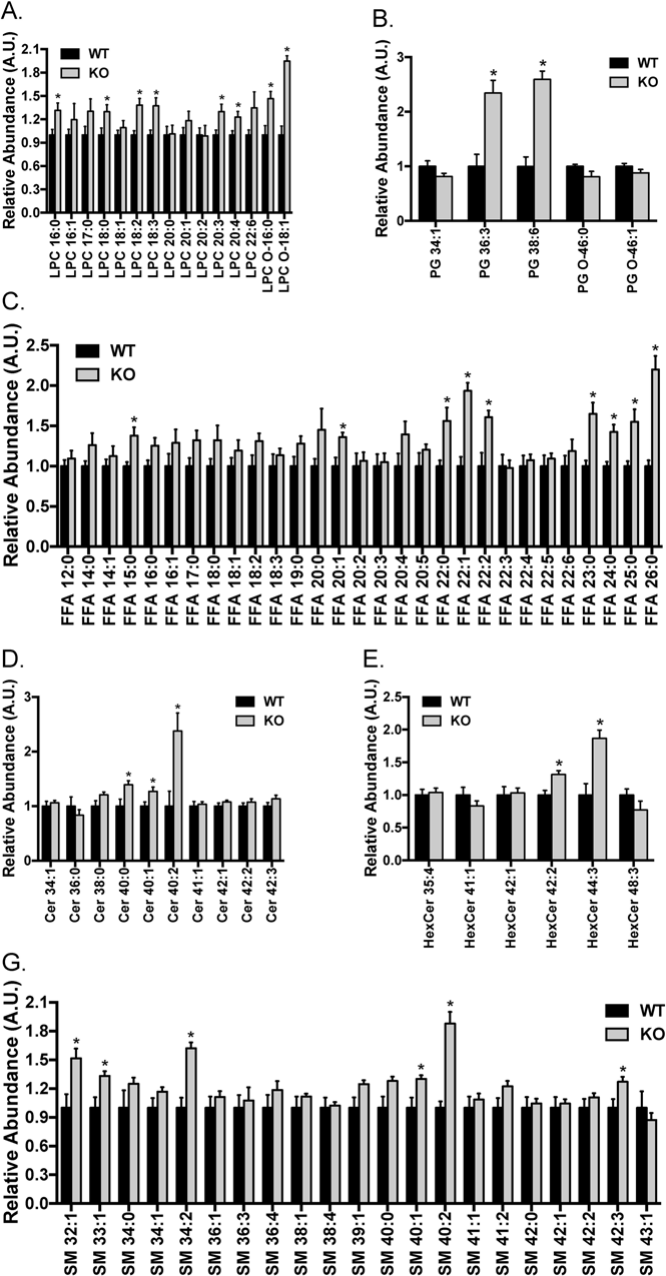
**Hepatic profiles of LPC, PG, FFA, Cer, HexCer and SM in the LDLR knockout rats.** Lipids were extracted from the liver, and LPC (A), PG (B), FFA (C), Cer (D), HexCer (E) and SM (G) species were determined via LC-MS. The levels of each lipid species in the wild-type rats were set to one, and their levels in the LDLR knockout rats were normalized against the respective wild-type values. The data are given as the mean±s.e.m., *P<0.05. WT: *n*=7; KO: *n*=5.

**Fig. 7.**
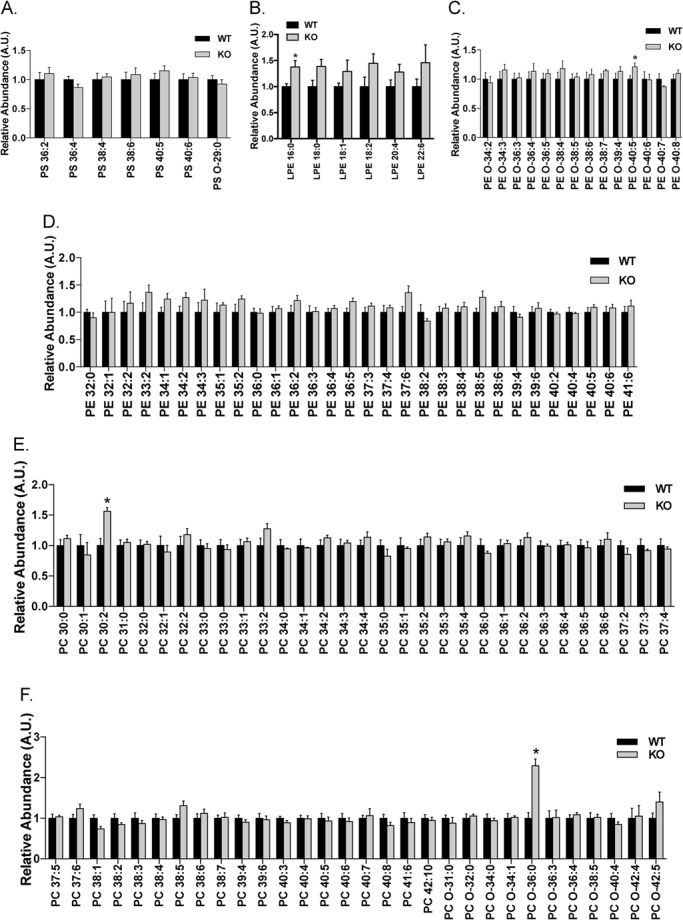
**Hepatic profiles of PS, LPE, PE and PC in the LDLR knockout rats.** Lipids were extracted from the liver, and PS (A), LPE (B), PE (C,D), PC (E,F) were determined via LC-MS. The levels of each lipid species in the wild-type rats were set to one, and their levels in the LDLR knockout rats were normalized against the respective wild-type values. The data are given as the mean±s.e.m., *P<0.05. WT: *n*=7; KO: *n*=5.

Major changes in the lipidomics of the LDLR knockout rats were observed in the lysophosphatidylcholines (LPC), free fatty acids (FFA) and sphingolipids (SL) including ceramides (Cer) and sphingomyelins (SM). Fifteen LPC species and five phosphatidylglycerol (PG) species were detected in the liver extracts and about half of them were significantly elevated in the liver of the LDLR knockout rats (Fig. 6A,B). Among the 29 FFA species detected, nearly a third of them were markedly increased in the liver of the LDLR knockout rats (Fig. 6C). Moreover, around a quarter of sphingolipid species displayed a significant increase in the liver of the LDLR knockout rats (Fig. 6D-G). In contrast, the levels of phosphatidylcholine (PC), lysophosphatidylethanolamine (LPE), phosphatidylethanolamine (PE) and phosphatidylserine (PS) remained largely unaltered in the liver of the LDLR knockout rats (Fig. 7A-F).

### Expression of key enzymes for hepatic lipid metabolism is upregulated in the LDLR knockout rats

**Fig. 8.**
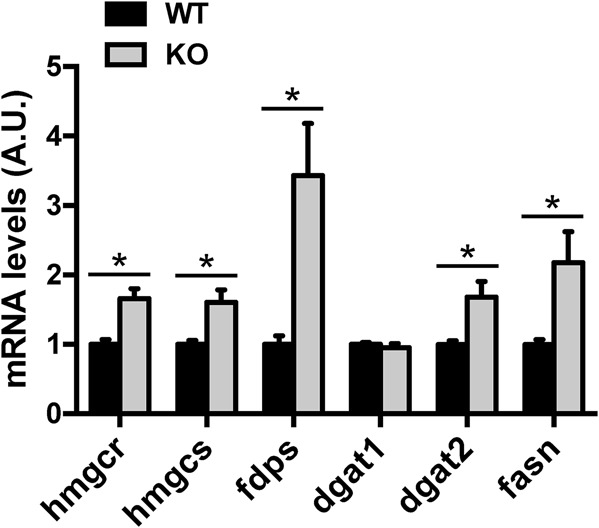
**Expression of key enzymes in lipid metabolism in the liver of the LDLR knockout rats.** The mRNA levels of *hmgcs*, *hmgcr*, *fdps*, *dgat*1, *dgat*2 and *fasn* in the liver were determined via Q-PCR. The data are given as the mean±s.e.m., *P<0.05. *n*=7-8.

To further understand how deletion of the LDLR impacts on lipid metabolism in the liver, we analyzed the expression of key enzymes involved. The expression of key cholesterol biosynthetic genes including 3-hydroxy-3-methylglutaryl coenzyme A synthase (HMGCS), 3-hydroxy-3-methylglutaryl coenzyme A reductase (HMGCR) and farnesyl diphosphate synthase (FDPS) was upregulated at the transcriptional level in the liver of the LDLR knockout rats (Fig. 8). The mRNA level of diglyceride acyltransferase 1 (Dgat1), an enzyme catalyzing the rate-limiting step in triglyceride synthesis, was normal in the liver of the LDLR knockout rats (Fig. 8). In contrast to Dgat1, the expression of Dgat2 was significantly upregulated at the mRNA level in the liver of the LDLR knockout rats (Fig. 8). The mRNA level of fatty acid synthase (Fasn) was also significantly higher in the liver of the LDLR knockout rats than that in the wild-type rats (Fig. 8). These data suggest that compensatory responses were induced to upregulate hepatic lipogenesis and cholesterol biosynthesis in the liver of the LDLR knockout rats.

## DISCUSSION

In this study, we generated a LDLR knockout rat that has a significant increase in plasma TC and TG, displayed an elevated plasma LDL-C and that can be used as a new animal model of hypercholesterolemia. The lipidomics analysis also revealed hepatic lipid signatures that associate with the LDLR deficiency in rats.

The LDLR mediates removal of VLDL, IDL and LDL from the plasma and thus regulates the plasma cholesterol level (Brown and Goldstein, 1986; Ishibashi et al., 1993). Lack of a functional LDLR in human patients causes hypercholesterolemia and accumulation of the plasma lipoproteins it transports (Goldstein and Brown, 1987). Eventually, deficiency of the LDLR results in fulminant atherosclerosis in human patients due to the massive elevation of plasma TC (600-1000 mg/dl) (Goldstein and Brown, 1989). In the Watanabe heritable hyperlipidemic rabbit, lack of a functional LDLR causes an 8- to 14-fold increase of plasma TC and also results in spontaneous development of aortic atherosclerosis (Watanabe, 1980; Yamamoto et al., 1986). Although deletion of the LDLR in mice increases plasma TC and LDL-C, the LDLR knockout mice do not develop atherosclerosis due to the relatively mild elevation of plasma TC (∼230 mg/dl) when fed on normal diet (Ishibashi et al., 1994a). Our LDLR knockout rats exhibit similar elevation of plasma TC and lipoprotein profiles as the LDLR knockout mice on normal chow diet (Table S2). Unlike humans or rabbits, mice produce two forms of apolipoprotein B in the liver, apoB-100 and apoB-48, with the latter as the dominant form (Higuchi et al., 1992). ApoB-48 containing chylomicron remnants can be efficiently cleared in an apoE dependent manner in the absence of the LDLR, which is mediated by the LDLR-related protein (LRP) (Ishibashi et al., 1994b; Rubinsztein et al., 1990). This apoE-mediated clearance of apoB-48 containing chylomicron remnants probably prevents the massive elevation of plasma TC in the LDLR knockout mice (Ishibashi et al., 1993, 1994a). ApoB-48 is also the major hepatic form of apolipoprotein B in rats (Reaves et al., 1996), which might also account for the lack of massive accumulation of plasma TC in the LDLR knockout rats similarly as in mice. It is also possible that other factors might restrict massive accumulation of plasma TC in small rodents such as mice and rats deficient of the LDLR. In line with this possibility, a xenograft mouse model using hepatocytes from a patient with familial hypercholesterolaemia caused by loss-of-function mutations in the LDLR only develops hypercholesterolaemia on cholesterol-rich high fat diet but not on normal chow diet (Bissig-Choisat et al., 2015). Nevertheless, probably due to the lack of the massive accumulation of plasma TC, we also did not observe arterial plague formation in the LDLR knockout rats fed on normal diet (data not shown) similarly as in the LDLR knockout mice fed on normal diet (Ishibashi et al., 1994a). Cholesterol-rich diet (1.25% cholesterol) can greatly increase plasma TC levels (>1500 mg/dl) and consequently results in fulminant aortic atherosclerosis in the LDLR knockout mice (Ishibashi et al., 1994a). Therefore, it deserves further investigation in future to find out whether such a cholesterol-rich diet can eventually lead to fulminant aortic atherosclerosis in the LDLR knockout rats. Despite the similarities in plasma TC and lipoprotein profiles between our LDLR knockout rats and the LDLR knockout mice, our LDLR knockout rats also displayed some features that are distinct from the LDLR knockout mice. For instance, the plasma TG levels were increased by 50% in the LDLR knockout rats (Table S2) while they remained normal in the LDLR knockout mice (Ishibashi et al., 1993). Interestingly, plasma TG levels were also significantly elevated in the Watanabe heritable hyperlipidemic rabbit (Watanabe, 1980).

The hepatic lipidomics study reveals no major change in glycerolipids (e.g. DAGs and TAGs) and glycerophospholipids (e.g. PCs, PEs and PSs). This is probably due to the active LRP pathway that rapidly removes apoB-48 containing chylomicron remnants from the plasma (Rubinsztein et al., 1990) and thus maintains relatively normal levels of CEs, glycerolipids and glycerophospholipids in the liver of the LDLR knockout rats. Another possibility is that LDLR deficiency caused a compensatory response in the liver of the LDLR knockout rats, in which hepatic lipogenesis and cholesterol biosynthesis are upregulated. In line with this possibility, the expression of key enzymes for lipogenesis and cholesterol biosynthesis including *hmgcs*, *hmgcr*, *fdps*, *dgat*2 and *fasn* were significantly increased in the liver of the LDLR knockout rats. Furthermore, nearly a third of FFA species were significantly elevated in the liver of the LDLR knockout rats, which also suggests an upregulation of hepatic lipogenesis. The hepatic lipidomics also reveals unexpected changes in LPCs and SLs. LPC can induce proinflammatory cytokines (Huang et al., 1999) and is involved in proatherogenic conditions (Quinn et al., 1988). Low LPC levels have been found to associate with an improved glucose tolerance and obesity-resistant phenotype in the mice lacking of group 1B phospholipase A2 that digests phospholipids into fatty acids and LPCs (Labonte et al., 2006, 2010). Similarly, certain SLs are elevated probably due to dysfunctional autophagy during pathologic conditions including aging, inflammation and metabolic diseases (Alexaki et al., 2014). Therefore, the increased LPCs and SLs may be indicative of proinflammatory conditions in the LDLR knockout rats and may affect the insulin sensitivity of the knockout rats. Although three out of four CEs were normal, in the rest of the CE species CE 20:4 was significantly increased in the liver of the LDLR knockout rat. Interestingly, CE 20:4 has recently been shown as a significant risk factor for the development of type 2 diabetes in women with a previous history of gestational diabetes mellitus (Lappas et al., 2015). In summary, this lipidomics study reveals a possible compensatory upregulation of hepatic cholesterol biosynthesis and lipogenesis in the absence of LDLR as indicated by elevated FFAs together with increased expression of cholesterol biosynthetic and lipogenic genes. Besides, it also suggests that deficiency of LDLR might promote hepatic inflammation as indicated by proinflammatory lipid species such as LPCs and SLs. These changes of hepatic lipid species revealed by lipidomic analysis warrant further investigation in future in order to fully understand their biological functions and implications.

In conclusion, the LDLR knockout rat generated in this study offers a new animal model of hypercholesterolemia, and the lipidomics analysis revealed hepatic lipid signatures that associate with deficiency of the LDL receptor in this rat model.

## MATERIALS AND METHODS

### Materials

All the restriction enzymes were purchased from Thermo Fisher Scientific (Waltham, MA, USA). Microcystin-LR was purchased from Taiwan Algal Science Inc (Taoyuan, Taiwan, China). Lipid internal standards of 1,2-dinonadecanoyl-sn-glycero-3-phosphocholine (PC 38:0); 1,2-diheptadecanoyl-sn-glycero-3-phosphoethanolamine (PE 34:0); 1-nonadecanoyl-2-hydroxy-sn-glycero-3-phosphocholine (LPC 19:0); N-lauroyl-D-erythro-sphingosylphosphorylcholine (SM 12:0); and N-heptadecanoyl-D-erythro-sphingosine (Ceramide 17:0) were from Avanti Polar Lipids, Inc. (Alabaster, Alabama, USA). Palmitic acid-16,16,16-d3 (FFA 16:0_d3), stearic acid-18,18,18-d3 (FFA 18:0_d3) and glyceryl tripentadecanoate (TAG 45:0) were purchased from Sigma-Aldrich (Munich, Germany). All other chemicals were from Sigma-Aldrich (St. Louis, Missouri, USA) or Sangon Biotech (Shanghai, China).

### Antibodies

The rabbit antibody against LDLR (Cat No. Orb39654) was from Biorbyt (Cambridge, UK), and the GAPDH antibody (Cat No. G8795) was from Sigma (St. Louis, Missouri, USA).

### Molecular biology

Molecular cloning was carried out using standard procedures. All DNA constructs were verified via DNA sequencing by Life Technologies (Shanghai, China). *In vitro* transcription of ZFN mRNA was performed using a mMESSAGE mMACHINE^®^ T7 Ultra Kit (Cat No. AM1345) from Ambion (Life Technologies/Thermo Fisher Scientific, Carlsbad, California, USA).

### Micro-injection and embryo implantation

The LDLR knockout rat was generated following the targeting strategy outlined in Fig. 1A. Briefly, the ZFN mRNAs targeting the *ldlr* gene were *in vitro* transcribed, and delivered into Sprague-Dawley rat zygotes via micro-injection with a dose of 50 pg of mRNAs per zygote. After micro-injection, rat zygotes were implanted into uterus of pseudopregnant female rats. Pups born from these implanted zygotes were screened for positive LDLR knockout animals.

### Rat housing, breeding and genotyping

All animal experiments were performed in accordance with experimental animal guidelines and regulations of Jiangsu Province, China. All animal studies, breeding and husbandry were approved by the Ethics Committee at Nanjing University. Animal experiments were carried out in a blinded manner. Wild-type Sprague-Dawley rats were bought from Vital River (Beijing, China). Rats were housed with a light:dark cycle of 12:12 h, and had free access to food and water.

Genotyping of mutant rats was performed via PCR amplification of target regions, cloning and sequencing the PCR fragments. The PCR primers for genotyping of mutant rats are as follows, 5′-TGCCTCTGGCTTCTGAGGACATC-3′ and 5′-TGGTGTGAGGGTCCATGTGGAAG-3′. PCR fragments were cloned into the pMD™18-T Vector (Cat No.6011, Takara), transformed into *E.coli*, and ten clones per rat were sent for sequencing.

### Blood chemistry and insulin analysis

Blood was collected from the retro-orbital venous plexus of overnight-fasted rats. Plasma glucose, free fatty acid, triglyeride and total cholesterol levels were measured using a Wako LabAssay glucose kit (298-65701), LabAssay NEFA kit (294-63601), LabAssay Triglyceride (290-63701) and LabAssay Cholesterol kit (294-65801) (Wako Chemicals USA, Inc.), respectively.

### Plasma lipoprotein analysis

Plasma lipoprotein profile was determined as previously described (Huang et al., 2015). Briefly, VLDL, LDL and HDL in rat plasma were separated through an AKATA Purifier 10 System (GE Healthcare Life Sciences, Chicago, USA). Cholesterol content in each eluate fraction was determined. Cholesterol in the fractions corresponding to each lipoprotein class detected via a UV detector at OD280 was summed up for quantification of VLDL-C, LDL-C and HDL-C.

### Liver TAG measurement

Liver TAG levels were determined as previously described (Norris et al., 2003). Briefly, liver chunks were taken from sacrificed rats and snap-frozen in liquid nitrogen before further analysis. Extraction of liver chunks was carried out in ethanolic KOH, and the resultant extracts were subsequently neutralized. Free glycerol resulted from breakdown of TAG was determined using the Free Glycerol Reagent (F6428, Sigma-Aldrich).

### Tissue lysis and protein measurement

Rat liver chunks were homogenized in lysis buffer using a Polytron homogenizer (Kinematica, Luzern, Switzerland) as previously described (Wang et al., 2013). Liver homogenates were lysed on ice for 30 min, and tissue debris was removed through centrifugation to obtain liver lysates. Protein concentrations of liver lysates were determined using Bradford reagent (Thermo Fisher Scientific).

### Immunoblotting

Immunoblotting was carried out as previously described (Wang et al., 2013). Briefly, proteins were denatured in Laemmli sample buffer, separated on SDS-PAGE gels, and subsequently immunoblotted onto nitrocellulose membranes. After immunoblotting, membranes were blocked in milk, and sequentially incubated with the indicated primary antibodies and horseradish-peroxidase-conjugated secondary antibodies (Promega). Chemiluminescence signals were detected using a gel documentation system (Syngene, UK) after membranes were incubated with ECL substrates (GE Healthcare, UK).

### RNA isolation and qPCR

After total RNA was isolated from liver samples using the TRIzol^®^ Reagent (Life Technologies), it was reverse-transcribed to synthesize cDNA with a PrimeScript^®^ RT reagent kit (DRR047A, TaKaRa). qPCR was performed using an Applied Biosystems^®^ StepOnePlus™ Real-Time PCR system (Life Technologies) and the primers listed in Table S3 to quantify expression levels of target genes.

### Liver lipid extraction and lipidomics

Lipidomics study was performed as previously described (Huang et al., 2013). Briefly, 10 mg wet liver tissue from liver chunks was weighed in a 2 ml microfuge tube with a steel bead in it. After addition of 400 μl cold methanol/water (3:1, v/v) containing eight lipid internal standards with appropriate concentrations, lipids were extracted through homogenization (at 20 Hz for 1 min), then 1 ml methyl tert-butyl ether (MTBE) was added and the mixture was shaken for 15 min. Subsequently, 300 μl of ultrapure water (18.2 MΩ-cm, TOC=6 ppb; Millipore, USA) was added to form a two-phase system. After vortexing for 30 s, the obtained mixture was left to stand for 10 min at 4°C. Liver lipid extracts were then deproteinized by centrifugation at 10,000 rpm (10,621 ***g***) for 10 min at 4°C. The resultant supernatants were lyophilized and stored in a −80°C freezer if needed. The freeze-dried lipid residues were resuspended in isopropanol/acetonitrile/water (30:65:5, v/v/v) and used for subsequent analysis via untargeted liquid chromatography (LC) coupled to a 5600 Triple time of flight-mass spectrometry (TripleTOF/MS) (TripleTOF™ 5600, AB Sciex). All lipids were analysed through an electrospray ionization-positive mode except FFAs that were analysed via an electrospray ionization-negative mode. The data matrix, including samples, identified lipid metabolites and corresponding concentrations were imported into the SIMCA-P program (Umetrics, Sweden) for univariate analysis. A partial least squares discriminant analysis after orthogonal signal correction (OSC-PLS-DA) was applied to the lipidomic data with unit variance scaling (Huang et al., 2013). Heat map analysis was carried out using a free software package available online at http://www.metaboanalyst.ca (version MetaboAnalyst 3.0).

### Statistical analysis

Data were analysed via Student's *t*-test, and differences were considered statistically significant at *P*<0.05.
